# Energetic and Spectroscopic Insights into the C_3_H_6_O_2_ Isomer Family for Astrochemical Purposes

**DOI:** 10.1021/acsearthspacechem.5c00291

**Published:** 2025-12-16

**Authors:** Alessandra Savarese, Silvia Alessandrini, Mattia Melosso, Gabriele Panizzi, Michela Nonne, Luca Bizzocchi, Cristina Puzzarini

**Affiliations:** † Dipartimento di Chimica “Giacomo Ciamician”, 657489 Università di Bologna, Via P. Gobetti 85, Bologna 40129, Italy; ‡ 9296 Scuola Superiore Meridionale, Largo San Marcellino 10, Naples 80138, Italy

**Keywords:** astrochemistry, minimum energy principle, computational protocol, C_3_H_6_O_2_ isomers, rotational spectroscopy, glycidol

## Abstract

A computational protocol based on the minimum energy principle has been applied to the C_3_H_6_O_2_ isomer family, providing accurate energetic hierarchies and spectroscopic parameters relevant to astrochemistry. The calculations predict propanoic acid as the most stable isomer, followed by methyl acetate, ethyl formate, 1-hydroxyacetone, 2- and 3-hydroxypropanal. The protocol delivers computed rotational spectroscopic parameters, and their accuracy has been benchmarked against literature results for seven C_3_H_6_O_2_ isomers and further validated through new high-frequency measurements of glycidol, c-C_2_H_3_O‑CH_2_OH. Its rotational spectrum has been recorded in the 65–120, 146–330, and 440–520 GHz ranges, extending the frequency coverage with respect to previous studies. The improved set of spectroscopic parameters for glycidol provides a basis for future radioastronomical searches in the interstellar medium. Furthermore, the benchmarking strategy establishes reliable uncertainties for the species not yet characterized in the laboratory.

## Graphical abstract

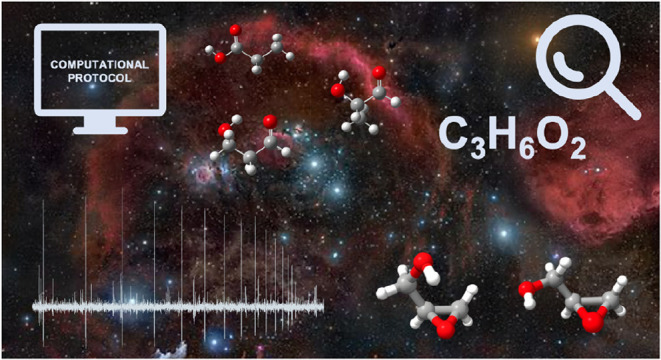

## 1 Introduction

Despite its harsh physical conditions, the interstellar medium (ISM) is characterized by a rich chemistry, with more than 340 species that have been identified to date.^1–3^ Interstellar molecules are mostly detected using radioastronomy: in line surveys, the rotational transitions of the species of interest are searched for using line catalogs as reference.^4^ The laboratory characterization is thus unavoidable. In this context, a possible strategy to identify potential candidates for astronomical detection is to focus on a suitable family of isomers and derive the most promising candidates to study in the laboratory. To do so, an empirical rule known as the Minimum Energy Principle (MEP) can be exploited. This states that (1) the most stable isomer of a given family is the most abundant in the ISM, and (2) the abundance ratio of two isomers of the same family depends on their energy difference.^5^ Thus, the MEP provides an empirical criterion to guide radioastronomical searches and laboratory efforts. However, some exceptions to this principle, i.e., some isomer families showing detectability trends that deviate from MEP predictions, have been reported in the literature.^5–12^ Some examples can be found in the C_3_H_2_O, C_2_H_2_N_2_, and C_2_H_5_O_2_N isomer families, where kinetic effects prevail over thermodynamic ones.^6–9^ Other causes of deviation from the MEP include desorption mechanisms from grain surfaces^5,13^ as well as spectral complexity^13^ and partition function effects.^12^ Nonetheless, the utility of the MEP is demonstrated by the studies reported in the literature on this topic.^14–16^ Building on the MEP, a computational protocol able to provide accurate energetic information and spectroscopic properties was also proposed by Alessandrini et al.,^17^ where the C_3_H_3_NO family of isomers was studied, suggesting cyanovinyl alcohol as potential interstellar molecule.

Here, the focus is on the C_3_H_6_O_2_ isomer family, which contains a large number of reasonable interstellar candidates characterized by a wide variety of functional groups. In particular, three members of the family have already been found in the ISM: ethyl formate,^18,19^ methyl acetate,^19^ and 1-hydroxyacetone.^20^ More recently, a tentative detection of 3-hydroxypropanal was also reported.^21^ Other species have been characterized in the laboratory but remain elusive in the ISM: propanoic acid,^22–25^ 2-hydroxypropanal,^26^ 2-methoxyacetaldehyde,^27,28^ 1,3-dioxolane,^29–32^ and glycidol.^33,34^ The family is also relevant for interstellar grains, as Wang et al.^35^ showed that the irradiation of low-temperature ices composed of methanol and acetaldehyde leads to formation of 1-hydroxyacetone, methyl acetate, 3-hydroxypropanal, and their enol tautomers.^35^ Additionally, the formation of 2- and 3-hydroxypropanal, ethyl formate, and 1,3-propenediol was observed upon irradiation of CO-ethanol interstellar ice analogs.^36^ These molecules could thus form in ices irradiated by galactic cosmic rays already at the prestellar stage, and then be released in the gas phase during the warm-up phase. All these species are also well suited for characterization by micro-, millimeter-, and submillimeter-wave spectroscopy due to their generally large dipole moment^28^ which results in a high intensity of the rotational transitions. This makes C_3_H_6_O_2_ isomers promising targets for future radioastronomical detections.^35^

While computational studies on the isomerization enthalpies and the relative energies of some of the molecular species belonging to the C_3_H_6_O_2_ isomer family are present in the literature,^16,35^ a comprehensive energetic investigation aimed at providing spectroscopic insights on the C_3_H_6_O_2_ isomers is still lacking. Moreover, some C_3_H_6_O_2_ species suffer from poor experimental characterization, preventing accurate knowledge of their rotational transitions, especially at high frequencies.^37^ This work will extend the experimental measurements on glycidol, whose rotational spectrum was recorded up to only 40 GHz in 1992.^33,34^ The choice of glycidol was three-fold. First, the molecule lacks an experimental characterization at high frequencies, more than other isomers of the family already studied in the literature. To a second instance, the molecule is strongly related to oxirane and propylene oxide, both species already observed in the ISM.^3^ Lastly, glycidol is an optimal choice for benchmarking theoretical calculations. In fact, the molecule is a rather rigid system owing to its intramolecular hydrogen bond. Hence, it is well suited to test the quality of molecular parameters derived in the framework of perturbation theory.

In Section 2, we present the computational protocol employed to study the C_3_H_6_O_2_ isomers, along with the details on the experimental setup used to derive high-frequency measurements of glycidol. In Section 3, the results are presented and are followed by a detailed discussion (Section 4) with respect to their astrochemical implications and future laboratory studies.

## 2 Methodology

As illustrated in Figure 1, the first step of the computational and experimental methodology employed in this work is the generation of all the isomers of the C_3_H_6_O_2_ family. Then, a computational protocol based on the MEP^17^ is applied with the aim of deriving the most promising interstellar candidates. The accuracy of the rotational spectroscopic data derived from the protocol is tested against: (i) the C_3_H_6_O_2_ isomers already studied in the literature and (ii) glycidol, purposely investigated in this work. For the latter, new experimental measurements are thus reported.

**Figure 1:**
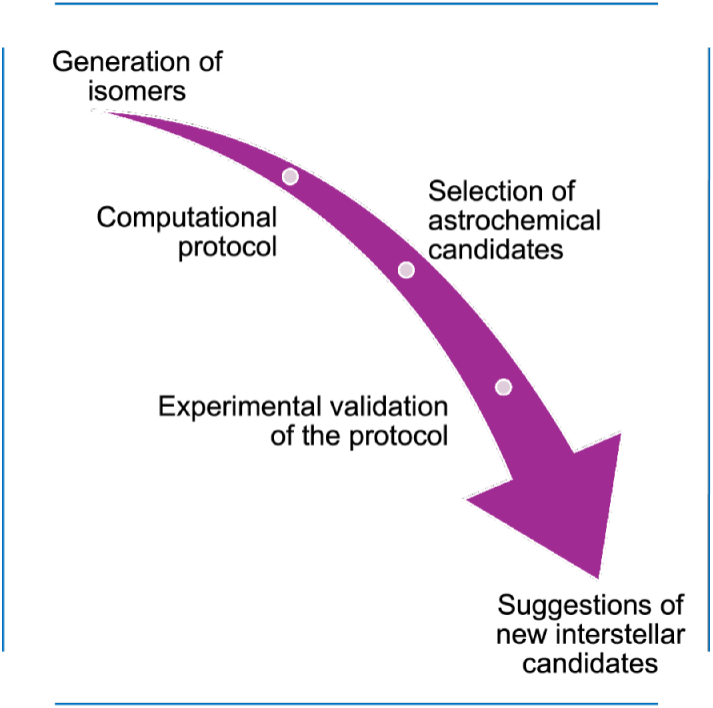
Schematic representation of the workflow employed in the present work to study the C_3_H_6_O_2_ isomer family.

### 2.1 Computational Details

The isomers of the C_3_H_6_O_2_ species were obtained by means of SciFinder-*n*.^38^ Here, the intrinsically less stable species (such as carbenes, zwitterionic structures, etc.) were discarded *a priori* as previously done in the literature.^17^ All density functional theory (DFT) calculations mentioned in the following were performed using the Gaussian16 suite of programs,^39^ while the CFOUR program package^40,41^ was used for the computations employing methods rooted in the coupled-cluster (CC) theory. An estimate of the computational cost (in terms of wall time) of each step of the protocol can be found in Table S1 of the Supporting Information (SI).

**Step 1: Preliminary Investigation.** Given the large number of species belonging to the C_3_H_6_O_2_ isomer family, this first step focused on generating and optimizing only one conformer for each possible structure using an affordable computational approach. In particular, a conformer for each isomer was generated randomly, since the difference in conformational energies for a given isomer is negligible at this stage. Step 1 of the protocol is, indeed, a screening step that aims to provide a first general idea of the energy scale. The equilibrium geometries (and related electronic energies) of the species were obtained by means of the B3LYP^42–47^ functional including Grimme’s D3 empirical correction with the BJ dumping function.^48^ This global hybrid functional is combined with the cc-pVDZ basis set,^49,50^ thus having (in short) the B3/DZ level of theory. To ensure that the stationary points located on the potential energy surface (PES) are minima, the corresponding Hessian matrix was evaluated, also obtaining the zero point energy (ZPE) correction within the harmonic approximation (hZPE). Using the B3/DZ level of theory, an energy scale was generated and only the isomers in the 0–200 kJ/mol energy range were retained for the next step.

**Step 2: Extension to the Conformers.** As in astrochemistry all the conformers of a species might be relevant, these have to be taken into account. This step has the specific aim of deriving all the possible conformers of the species lying in the 0–200 kJ/mol energy range as obtained from Step 1. The conformers were generated by means of CREST,^51^ using the GFN Force Field.^52^ In some cases, the program did not provide all the possible conformers and the missing ones were manually generated. Then, the conformers went through the same calculations of Step 1 and those in the 0–150 kJ/mol energy range moved on to Step 3.

Step 1 and 2 of the present protocol can be considered a prescreening of the species before moving forward to Step 1 of the protocol developed by Alessandrini et al.^17^ This adjustment was made to treat isomer families comprising of a large number of isomers, with each isomer being characterized by a significant number of conformers. Thus, the prescreening is carried out using a lower level of theory than Step 1 of the protocol by Alessandrini et al.^17^ by computing geometries and energies at the B3/DZ level of theory.

**Step 3: Geometry and Energy Improvement.** To obtain more reliable results, the level of theory was improved by exploiting a double-hybrid functional in conjunction with a partially augmented triple-ζ basis set. In more detail, the retained species from Step 2 were reoptimized and their harmonic force field was recomputed using the revDSDPBEP86/jun-cc-pVTZ^53–56^ level of theory, still including the D3 empirical correction and the BJ dumping function. This level is shortly denoted as revDSD/junTZ in the following. A third energy scale (hZPE corrected) was then constructed, and the 15 most stable species (up to 100 kJ/mol in the relative electronic energy scale) advanced to Step 4.

Step 3 of the present protocol aims at reconnecting to Step 1 of the protocol by Alessandrini et al.,^17^ by recomputing all energies and geometries at the revDSD/junTZ level of theory.

**Step 4: Final Geometry and Energy Refinement.** Step 4 of the present protocol corresponds to the third step of the protocol by Alessandrini et al.^17^ In particular, this step improved the molecular structures and the energies of the 15 most stable species by exploiting a composite scheme rooted in CC theory. The so-called CCSD­(T)/CBS+CV approach was employed in the final geometry and energy refinement. This composite scheme takes into account the extrapolation to the complete basis set (CBS) limit and the effects of core–valence (CV) correlation. The CCSD­(T)/CBS+CV energy (*E*(CBS+CV)) is defined as

1 $$E\left(\mathrm{CBS}\text{+}\mathrm{CV}\right)=E_{\infty }^{\mathrm{HF}‐\mathrm{SCF}}+\Delta E_{\infty }^{\mathrm{CCSD}\left(T\right)}+\Delta E_{\mathrm{CV}}$$

Here, $E_{\infty }^{\mathrm{HF}‐\mathrm{SCF}}$ and $\Delta E_{\infty }^{\mathrm{CCSD}\left(T\right)}$ are the extrapolations to the CBS limit of the HF-SCF energy and the CCSD­(T) correlation energy within the frozen-core (fc) approximation, respectively.^57,58^ The $E_{\infty }^{\mathrm{HF}‐\mathrm{SCF}}$ contribution is obtained using the three-parameter exponential model by Feller.^59^ This requires three energy computations, that have been carried out using the cc-pVTZ, cc-pVQZ, and cc-pV5Z basis sets.^49^ The $\Delta E_{\infty }^{\mathrm{CCSD}\left(T\right)}$ term is evaluated with the *n* ^–3^ formula by Helgaker et al.^60^ This formula requires two energy calculations. In this case, the cc-pVTZ and cc-pVQZ basis sets^49^ were used. Finally, Δ*E*_CV_ introduces the inner-shell electrons’ correlation as the difference between all-electron (ae) and fc calculations.^57,58^ In this work, the Δ*E*_CV_ term was computed with the cc-pCVTZ basis set.^61^

The CCSD­(T)/CBS+CV geometries are computed by building and then minimizing an energy gradient based on the CCSD­(T)/CBS+CV energy:^57,58^

2 $$\frac{dE_{\mathrm{CBS}+\mathrm{CV}}}{dx}=\frac{dE_{\infty }^{\mathrm{HF}‐\mathrm{SCF}}}{dx}+\frac{d\Delta E_{\infty }^{\mathrm{CCSD}\left(T\right)}}{dx}+\frac{d\Delta E_{\mathrm{CV}}}{dx}$$

The CCSD­(T)/CBS+CV equilibrium energy was then corrected for the anharmonic ZPE (aZPE) by computing the anharmonic force field (AFF) at the revDSD/junTZ level of theory.^48,53–56^ From these data, a final energy scale was obtained for the 15 most stable species.

At the end of this step, all the relevant rotational spectroscopic information for the 15 most stable C_3_H_6_O_2_ isomers was also obtained. Within vibration perturbation theory to second order (VPT2),^62^ the rotational constants of the vibrational ground state (*B*_0_) are defined as

3 $$B_{0}^{\gamma }=B_{\mathrm{eq}}^{\gamma }+\Delta B_{\mathrm{vib}}^{\gamma }$$

where $B_{\mathrm{eq}}^{\gamma }$ is the equilibrium rotational constant, with *γ* = *a*, *b*, *c* referring to the principal inertia axis (with the rotational constants being denoted as *A*, *B*, and *C* if *γ* = *a*, *b* and *c*, respectively). This equilibrium term is straightforwardly obtained from the CCSD­(T)/CBS+CV equilibrium structure. $\Delta B_{\mathrm{vib}}^{\gamma }$ denotes the vibrational correction to the equilibrium rotational constant and it is obtained from the VPT2 analysis applied to the revDSD/junTZ AFF.^63^ Furthermore, AFF calculations also give access to the quartic and sextic centrifugal distortion constants. Our revDSD/junTZ calculations also provide the molecular dipole moment components.

### 2.2 Experimental Approach

As mentioned in the Introduction, glycidol is the C_3_H_6_O_2_ isomer chosen to test our protocol as its spectrum was measured in the literature only up to 40 GHz. In fact, extending measurements to higher frequencies provides more accurate data for astronomical detections and also a more accurate and complete data set of rotational parameters to compare with our theoretical results.

Experimentally, the rotational spectrum of glycidol was recorded in the 65–120 GHz, 146–330 GHz, and 440–520 GHz frequency ranges. The glycidol sample (96% purity) was purchased from Merck and used without further purification. Measurements were performed using the millimeter-wave frequency-modulation spectrometer recently described by Claus et al.^64^ Shortly, a W-band Signal Generator was used to generate millimeter-wave radiation in the 75–110 GHz frequency range. Higher frequencies were obtained via active and passive multipliers. Band-adapted Schottky-barrier diodes were used as detectors. The radiation is sine-wave modulated at 16.67 kHz and then demodulated at 2*f*. The recorded signals are thus the second derivative of the actual absorption profile.

The measurements were performed by flowing glycidol vapors through the 3 m-long absorption cell kept at a pressure of ∼10 μbar. To maintain a steady flow, the sample was heated at 60 °C. The measurement uncertainty on the line positions ranges from 20 kHz to 60 kHz, depending on the frequency region.

## 3 Results

Due to the two-fold nature of this work, the presentation of the results follows the methodology introduced above: computational results are first addressed, followed by consideration of experimental results. In the following, each isomer is denoted with a number, while conformers of a given isomer are distinguished by a letter. The association between this “letter-number” denomination and the molecular structure can be found in Table S2 of the SI.

### 3.1 Computational Results

**Step 1: Preliminary Investigation.** The preliminary energetic investigation of Step 1 was performed on 37 isomers, resulting in the relative energy scale depicted in Figure 2 (see also Table S2 of the SI). The most stable isomer at this level of theory is propanoic acid (**2**), which was thus used as reference for the energy scale. Only two other isomers lie within 100 kJ/mol above **2**, methyl acetate (**3**) and ethyl formate (**4**).

**Figure 2:**
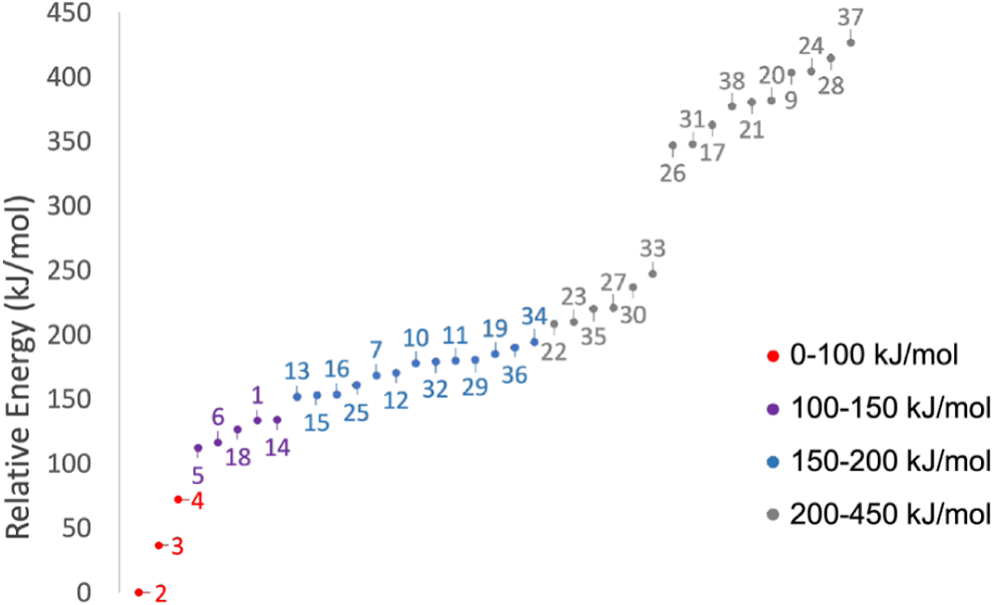
hZPE-corrected B3/DZ relative energies of the 37 C_3_H_6_O_2_ isomers as obtained from Step 1. The reference energy is that of isomer **2** (equilibrium energy = −268.427789 *E*_*h*_ and hZPE correction = 0.090026 *E*_*h*_).

The isomers in the 100–150 kJ/mol energy range are **5** (1-hydroxyacetone), **6** (2-hydroxypropanal), **18** (1-propene-1,1-diol), **1** (3-hydroxypropanal), and **14** ((1Z)-propene-1,2-diol). The 150–200 kJ/mol range features the presence of several propendiol species: **15** ((1E)-propene-1,2-diol), **16** (2-propene-1,1-diol), **12** (2-propene-1,2-diol), **10** ((1E)-propene-1,3-diol), and **11** ((1Z)-propene-1,3-diol). According to Wang et al.,^35^ several of these species (**15**, **12**, **10**, and **11**) might be present in interstellar ices and could be good candidates for astronomical detection. Some cyclic structures also lie in the 150–200 kJ/mol energy range (**25**, **32**, **29**, **36,** and **34**). The remaining 19 species are very high in energy (in the 200–450 kJ/mol energy range), and were thus excluded in the next step.

**Step 2: Extension to the Conformers.** By taking into account all the conformers of the 18 isomers retained after Step 1, the total number of species becomes 97. Their relative energy scale including hZPE (B3/DZ level) is detailed in Table S2 of the SI and portrayed in Figure S1 of the SI. Compared to the previous step, the 0–100 kJ/mol energy range contains one more species, i.e., the most stable conformer of 1-hydroxyacetone (**5a**), which is characterized by an intramolecular hydrogen bond. Similarly, one conformer of 3-hydroxypropanal (**1c**) is now more stable than 1-propene-1,1-diol (**18**). It is also possible to notice how the inclusion of conformers resulted in several changes in the 100–200 kJ/mol energy range.

**Step 3: Geometry and Energy Improvement.** The 40 conformers in the range 0–150 kJ/mol of the energy scale defined above were reoptimized using the revDSD/junTZ level of theory. This level of theory was used to also derive a new hZPE. The results of this level of theory are displayed in Figure 3 and detailed in Table S3 of the SI. In the 0–100 kJ/mol range, the positions of the **4a**–**4b** and **5d**–**5c** conformers pairs are swapped compared to the previous level of theory. Furthermore, one conformer of 2-hydroxypropanal (**6d**) lowers in energy, entering the 0–100 kJ/mol range. The 100–150 kJ/mol energy range is characterized by several changes in the energy scale. However, if one takes into consideration the relative order of the isomers (picking the most stable conformer for each isomer), such order remains unchanged. The only exception is (1*Z*)-propene-1,3-diol (**11**) that becomes the last in the energetic stability, preceded by (1*E*)-propene-1,2-diol (**15**). Using the revDSD/junTZ level of theory, two conformers (**1m** and **18e**) are no longer present in the potential energy surface, emphasizing the shortcoming of the B3/junDZ PES. As a matter of fact, B3/junDZ provides wrong orders between conformers. Therefore, while being a useful tool for the preliminary screening of PESs, it should not be employed as a reliable level of theory in conformational studies.

**Figure 3:**
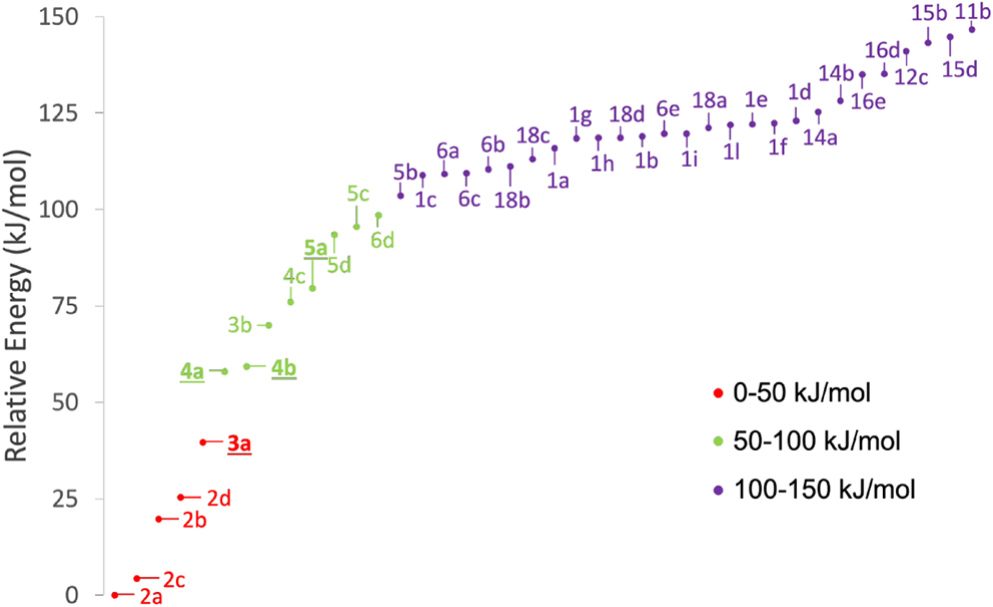
hZPE-corrected revDSD/junTZ relative energies of the 40 C_3_H_6_O_2_ conformers considered in Step 3. The energy of **2a** is the reference (equilibrium energy = −268.0460105 *E*_*h*_ and hZPE correction = 0.0908268 *E*_*h*_). The species underlined and highlighted in bold have already been detected in the ISM.

**Step 4: Final Geometry and Energy Refinement.** In this step, the geometries and energies are further improved for the 15 most stable conformers using the CCSD­(T)/CBS+CV level of theory and computing revDSD/junTZ AFFs. Figure 4 shows the final relative energetic scale. It can be seen that, when moving from the third to the fourth step of the protocol, the only exchange is between the **4c** and **5a** conformers, with the latter becoming lower in energy than the former. Additionally, Figure 4 shows that including anharmonicity effects in the ZPE correction (aZPE) has a minor effect on the relative energies, leaving the energy scale unaltered with respect to the inclusion of the hZPE. It is interesting to note that the energy difference between two conformers of ethyl formate, namely **4a** and **4b**, which is expected to be 0.78 ± 0.25 kJ/mol based on the experimental determination by Riveros and Wilson,^65^ is predicted to be 1.2 kJ/mol, thus slightly above the upper limit of the literature datum.

**Figure 4:**
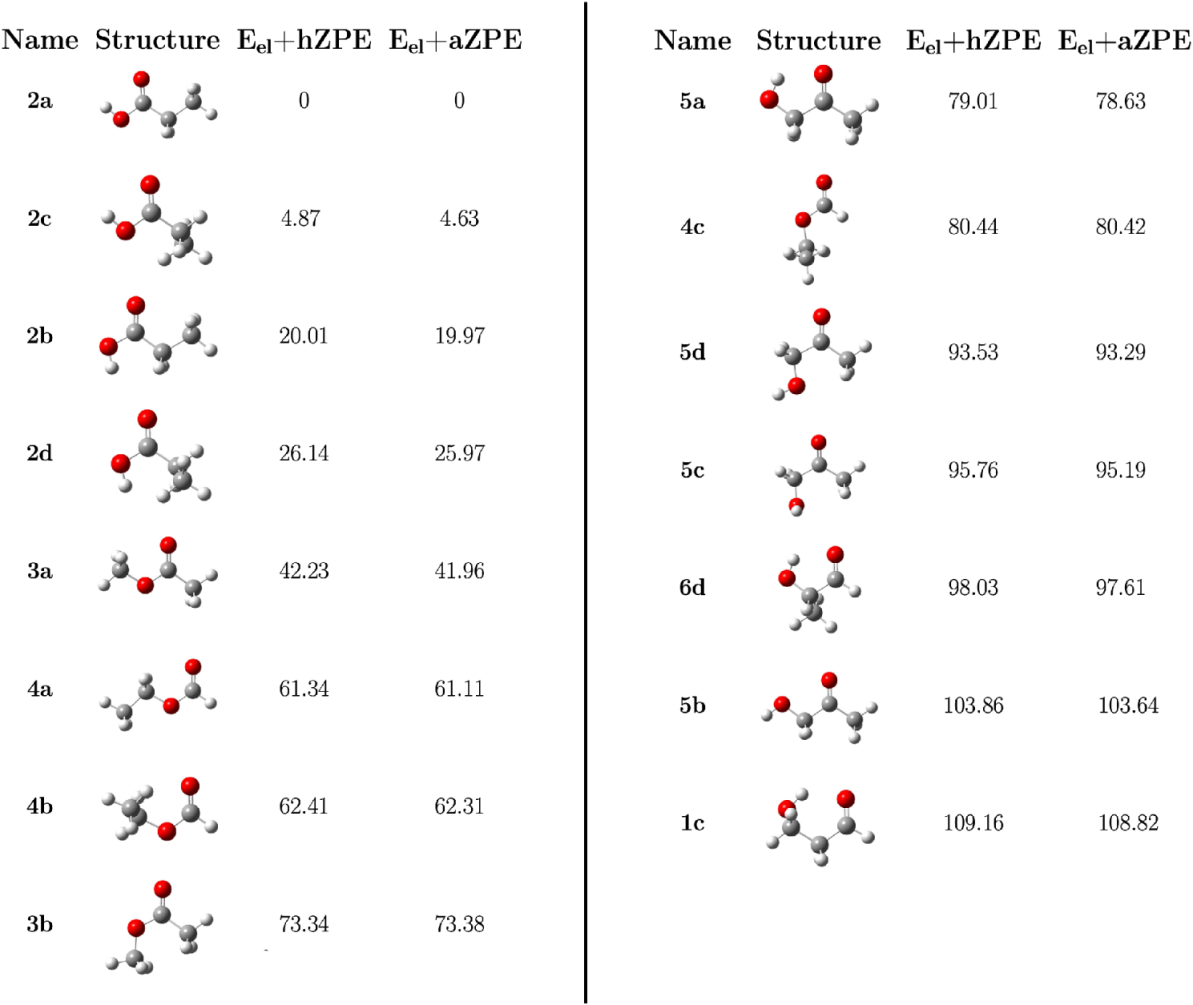
Final hZPE- and aZPE-corrected relative energetic scales (kJ/mol) for the 15 most stable C_3_H_6_O_2_ conformers (Step 4). The CCSD­(T)/CBS+CV electronic energy *E*_el_ is corrected for both hZPE and aZPE contributions, computed at the revDSD/junTZ level of theory. The energy of **2a** is the reference (equilibrium energy = −268.3860490 *E*_*h*_ and aZPE correction = 0.0896949 *E*_*h*_).

In addition to the relative stability of the C_3_H_6_O_2_ isomers, the magnitude of their dipole moment needs also to be taken into account. As a matter of fact, the intensity of the rotational transitions for a given molecular species not only depends on its abundance (linked to the energetic stability according to the MEP), but also on its dipole moment (whose components determine the intensity of rotational transitions). Nonetheless, while the following discussion focuses on the energetic stability and the magnitude of the dipole moment, one should remember that the detectability of interstellar species is also influenced by other factors (such as kinetic^6–9^ and partition function^12^ effects, spectral complexity,^13^ and desorption from grain surfaces).^5,13^

Figure 5 plots the total electric dipole moment (at the revDSD/junTZ level of theory) against the relative energy of the species for the first 15 conformers. The species in blue in Figure 5 are the conformers already detected in the ISM, namely: **3a** for methyl acetate, **5a** for 1-hydroxyacetone, and **4a** and **4b** for trans- and gauche-ethyl formate.^18–20^ It is interesting to note that the species observed are not the most stable within the family, indeed being located in the 40–80 kJ/mol range with respect to the most stable conformer, but they possess a quite large dipole moment (greater than 3 debye). Instead, the conformers **2a** and **2c** of propanoic acid, which are the most stable species within the family, are characterized by the smallest dipole moment. The two forms of propanoic acid **2b** and **2d** have instead a much larger dipole moment and are located only slightly higher in energy, still lying lower in energy with respect to the species already observed in the ISM. Therefore, they can be considered good targets for astronomical searches although their spectroscopic characterization is lacking. Other interesting species that stand out from this graph are **3b** and **4c**, that correspond to higher-energy conformers, with larger dipole moment, of species already observed. Furthermore, according to Figure 5, molecules relevant to astrochemistry are **6d** and **1c**, i.e., the most stable conformers of 2- and 3-hydroxypropanal, respectively. The confirmation that these species are of interstellar interest is provided by the numerous (yet unfruitful) searches for 2-hydroxypropanal toward many interstellar sources,^26^ as well as by the recent spectroscopic study and tentative detection of 3-hydroxypropanal, that was published while this paper was in preparation.^21^

**Figure 5:**
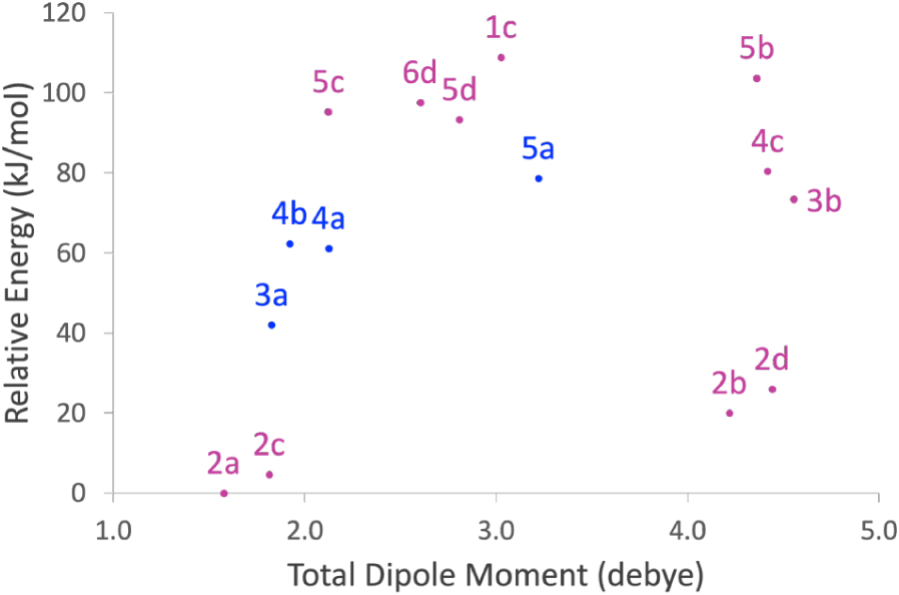
Total electric dipole moment (D) vs relative energy (kJ/mol) for the first 15 conformers of the C_3_H_6_O_2_ isomers. The equilibrium energy is computed with the CCSD­(T)/CBS+CV composite scheme, while the dipole moment and aZPE correction are at the revDSD/junTZ level of theory. The species in blue have already been detected in the ISM.

The results on the spectroscopic parameters obtained in this step and their accuracy will be discussed in Section 4. Before that, the outcomes of the new experimental measurements on glycidol are presented.

### 3.2 Experimental Results

Glycidol (labeled as **30** in the computational protocol) ranked 26th in the relative energy scale obtained in Step 1 of the aforementioned protocol. Lying in the 200–450 kJ/mol energy range, it did not move forward to Step 2. It was, however, chosen to validate experimentally the accuracy of the rotational constants obtained through the computational protocol for the three reasons mentioned in the Introduction.

Glycidol has two conformers that differ in the orientation of the hydroxyl group,^34^ as shown in Figure 6. The most stable is denoted as inner, while the second conformer (outer) is located 3.6(4) kJ/mol above the former, according to experimental measurements.^34^ Thus, at room temperature, the expected population is 83% inner and 17% outer and both species are observable in the spectrum. Both inner and outer glycidol are classified as asymmetric rotors, with computed Ray’s asymmetry parameters *k* of −0.902 and −0.934, respectively.

**Figure 6:**
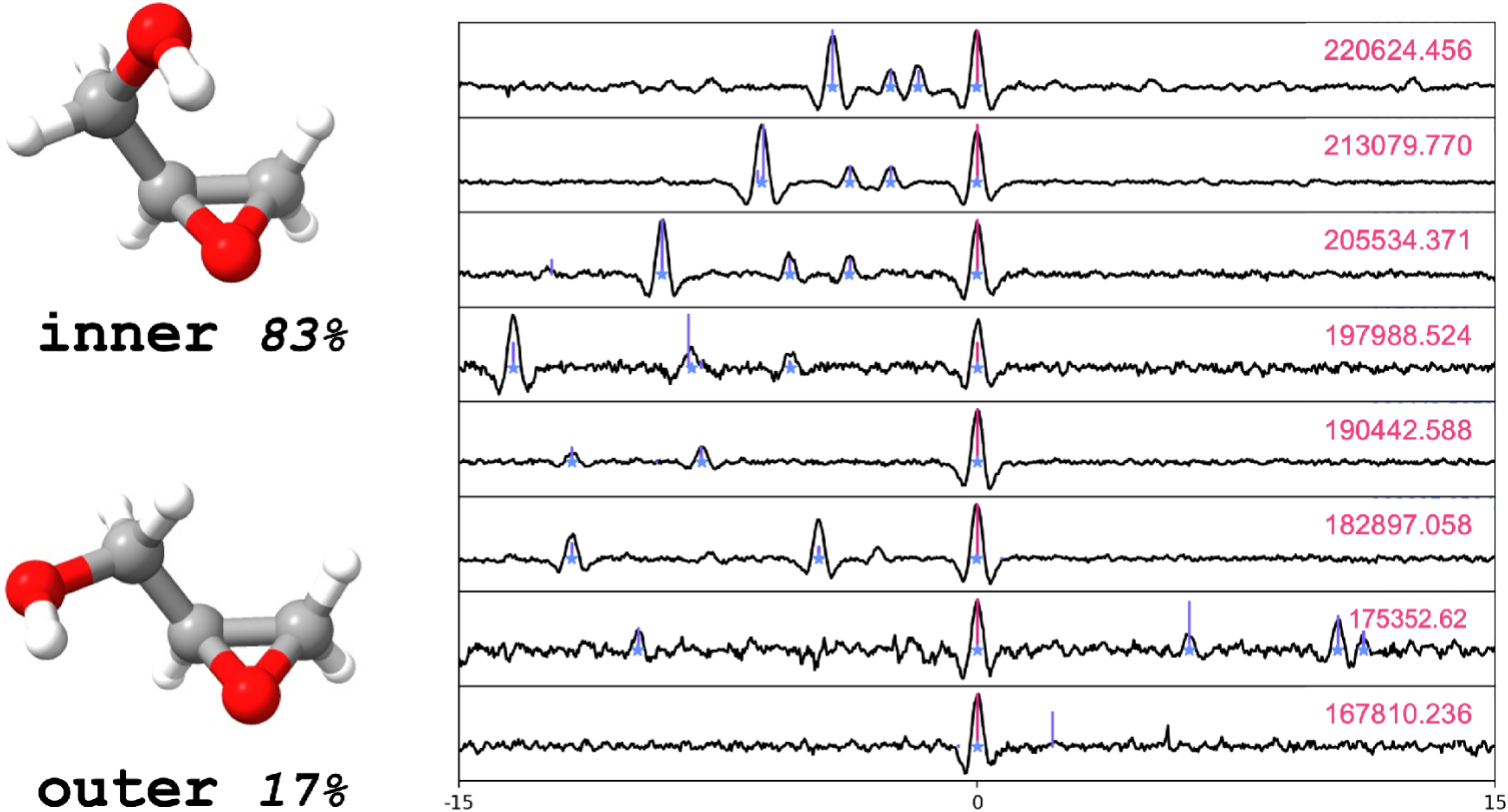
On the left, graphical representation of the inner and outer conformers of glycidol. On the right, Loomis–Wood plots obtained with the LLWP code^66^ of some *b*-type transitions (in pink) of the inner conformer. The related frequencies (on the right) are in MHz.

The assignment of the experimental rotational spectra was guided by a simulated spectrum based on the low-frequency transitions reported by Marstokk et al.,^34^ that were refitted in the S-reduction using the SPFIT program.^67^ In most cases the simulated transitions showed little (but non-negligible) deviation from the experimental ones; in some cases, deviations of some tens of MHz were observed. For the inner conformer, the line assignment started from the *b*-type transitions. These were mainly of *R*-branch, but several *Q*-branch lines could be assigned as well. In addition, distinguishable *R*-branch *c*-type transitions were also assigned, for a total of nearly 3700 new transitions.

A similar procedure was followed for the outer conformer. In this case, the most intense transitions are the *a*- and *b*-type. In particular, *R* branches were assigned for both *a*- and *b*-type transitions, while *Q*-branches were observed only for the *b*-type. As expected, the lines of the outer conformer are less intense compared to those belonging to the inner species, due to its lower abundance.

The S-reduced rotational spectroscopic parameters, resulting from fitting the assigned transitions and those reported by Marstokk et al.,^34^ for the inner and outer conformers of glycidol are summarized in Table 1, along with the theoretical ones obtained with the present computational protocol. The comparison of the experimental and computational parameters obtained in this work for inner and outer glycidol will be addressed in Section 4.

**Table 1:** Spectroscopic Parameters (*S*-Reduction) of the Inner and Outer Conformers of Glycidol

inner			outer		
Constant	Unit	Experiment^a^	Theory^b^	Experiment^a^	Theory^b^
*A*_0_	MHz	10,347.86548(9)	10,365.331	13,857.0864(2)	13,888.334
*B*_0_	MHz	4102.38046(3)	4099.420	3420.49834(5)	3418.266
*C*_0_	MHz	3781.93270(3)	3779.743	3065.88426(5)	3065.880
*D*_*J*_	kHz	2.50514(1)	2.543	2.30591(3)	2.301
*D*_JK_	kHz	–2.16006(7)	–2.353	–15.0687(2)	–15.04
*D*_K_	kHz	5.7912(2)	5.786	54.7590(8)	53.82
*d*_1_	kHz	–0.314787(3)	–0.3253	–0.403240(1)	–0.3995
*d*_2_	kHz	0.060685(1)	0.05674	–0.013772(3)	–0.01308
*H*_*J*_	mHz	–2.538(1)	–2.745	5.498(8)	6.979
*H*_*JK*_	mHz	7.12(2)	8.736	–73.88(7)	–91.06
*H*_*KJ*_	mHz	–11.03(8)	–1.468	157.6(4)	236.7
*H*_*K*_	mHz	24.0(1)	26.57	96.4(8)	65.26
*h*_1_	mHz	–0.2985(3)	–0.3832	1.395(3)	1.845
*h*_2_	mHz	0.1608(2)	0.1385	–0.017(1)	–0.01316
*h*_3_	mHz	0.01296(7)	0.009151	0.01155(7)	0.01203
*L*_*J*_	μHz	-	-	0.0203(6)	-
*L*_*JJK*_	μHz	–0.017(2)	-	–0.323(7)	-
*L*_*JK*_	μHz	–0.18(1)	-	–1.52(7)	-
*L*_*KKJ*_	μHz	0.48(3)	-	–3.8(3)	-
*l*_1_	μHz	-	-	0.0079(3)	-
*l*_2_	μHz	-	-	0.0015(1)	-
μ_a_	D	0.61(2)	0.63	1.25(6)	1.31
μ_b_	D	1.20(9)	1.23	1.650(1)	1.51
μ_c_	D	0.51(12)	0.78	0.154(2)	0.19
Lines^c^		3750/5935		2795/4853	
Max. *J*, *K*_*a*_		99, 46		89, 39	
rms	kHz	39.2		39.9	
St. Dev.		0.97		1.04	

a The standard errors as provided by PIFORM (Z. Kisiel, PROSPE—Programs for ROtational SPEctroscopy, https://info.ifpan.edu.pl/~kisiel/prospe.htmhttp://info.ifpan.edu.pl/~kisiel/prospe.htm) are indicated in parentheses. Experimental dipole moment values are from Marstokk et al.^34^

b Equilibrium rotational constants computed at the CCSD­(T)/CBS+CV level of theory and augmented by revDSD/junTZ vibrational contributions. Centrifugal distortion constants computed at the revDSD/junTZ level of theory.

c Distinct frequencies included in the fit/total number of transitions. It includes 73 transitions for inner and 66 for outer from Marstokk et al.^34^

The A-reduction was also tested for the fit of the rotational spectroscopic parameters of inner and outer glycidol. It should be noted that the quality of the fit performed with the S-reduction is comparable to the one carried out with the A-reduction, provided that three more parameters are added to the latter fit. The A-reduced experimental parameters are reported in Table S4 of the SI, together with the parameters obtained by Marstokk et al.,^34^ so as to allow a comparison between the two sets. Such a comparison reveals that the accuracy on the Δ_*JK*_, δ_*K*_, and Φ_*J*_ parameters of the inner conformer was improved by 1 order of magnitude. For the same conformer, the ϕ_*J*_, ϕ_*JK*_, ϕ_*KJ*_, ϕ_*K*_, Λ_*J*_, Λ_*JJK*_, Λ_*JK*_, Λ_*KKJ*_, Λ_*K*_, and λ_*J*_ parameters were experimentally determined for the first time. With regard to the outer conformer, the accuracy on *C*_0_, Δ_*J*_, Δ_*JK*_, Δ_*K*_, and δ_*K*_ was improved by 1 order of magnitude, while the Φ_*JK*_, Φ_*K*_, ϕ_*J*_, ϕ_*JK*_, ϕ_*K*_, Λ*_J_*, Λ_*JJK*_, Λ_*JK*_, Λ_*KKJ*_, λ_J_, and λ_*JK*_ parameters were determined for the first time. These new improved experimental spectroscopic data will be useful for guiding future radioastronomical searches in the millimeter- and submillimeter-wave regions.

## 4 Discussion and Conclusions

After the presentation of both the computational and experimental results, here we focus on an analysis aiming to assess the accuracy and reliability of our computational protocol as well as on a discussion of the astrochemical implications.

In this work, the C_3_H_6_O_2_ isomer family was theoretically characterized from a structural and energetic point of view, by means of a computational protocol based on the MEP. Such computational protocol was derived from the one developed by Alessandrini et al.,^17^ with adjustments made to treat isomer families comprising of a significant number of species. This investigation allowed us to draw some conclusions on which members of the family have the highest probability of being found in the ISM. According to the final relative energy scale, the most stable C_3_H_6_O_2_ species is the cis conformer of propanoic acid (**2a**). This has been claimed as relevant for interstellar chemistry for a long time;^25^ however, none of the searches have been fruitful. Indeed, propanoic acid represents one exception to the MEP, as several other isomers of the family are detected in space. This is not strange as acetic acid, the most stable isomer of the (smaller) C_2_H_4_O_2_ family, is also an exception to the MEP. Indeed, CH_3_COOH has an abundance 10 times lower than that of methyl formate, which is located about 70 kJ/mol higher energy. The lower abundance of carboxylic acids in the ISM has been ascribed to the fact that these species are strongly bounded to ice-surfaces.^5^ Furthermore, our protocol suggests that **2b** and **2d** are more likely observable in the ISM. Indeed, these species have a larger dipole moment than **2a** and **2c**, still lying lower in energy than the C_3_H_6_O_2_ isomers that have already been detected. To date, astronomical searches of **2b**, **2c,** and **2d** have been prevented by the lack of suitable experimental spectroscopic data. On one hand, their spectral characterization by means of room-temperature measurements is likely to be hindered by the congested nature of the rotational spectrum of **2a** in the millimeter-/submillimeter-wave regime at room temperature (as reported by Ilyushin et al.^25^). As a matter of fact, the presence of both low-energy vibrational modes and the additional methyl torsion splittings^25^ would prevent the straightforward identification of the spectral lines of the higher-energy conformers. On the other hand, the laboratory characterization of **2b**, **2c,** and **2d** through supersonic jet experiments might be hampered by relaxation phenomena; however, spectral measurements in such conditions should be feasible if relaxation barriers toward **2a** are sufficiently high. Therefore, it is particularly important to explore the conformational PES of propanoic acid at a suitable and reliable level of theory.

In 1975, two works targeting propanoic acid have been published on conformer **2a**.^68,69^ A subsequent paper on the spectroscopic characterization of the gauche conformer **2c** was announced by the author, but actually never published. Thus, **2c** could be an interesting target for a future spectroscopic characterization. As a matter of fact, the idea that these species are present in the ISM is endorsed by the recent detection of trans-methyl formate,^70^ this conformer lying about 25 kJ/mol above the most stable cis form.^70^ However, the reported *cis*/*trans* isomeric ratios for methyl formate is quite far from the expected thermodynamic ratio. Furthermore, the *Aa* conformer of n-propanol was observed in the ISM, this species being 0.3 kJ/mol less stable than the *Ga* conformer. In this case, the reported *Ga*/*Aa* abundance ratio of 1.64 toward G+0.693 is consistent with the two conformers being in thermodynamic equilibrium.^71^

Next in the energy scale, above the conformers of propanoic acid, we find **3a** (methyl acetate), **4a** and **4b** (*cis*- and *gauche*-ethyl formate) and **5a** (1-hydroxyacetone). These species have all been identified in the ISM, thus suggesting that **3b**—located in energy between **4b** and **5a**—might also be present. The next isomers in the energy ladder are **6d** and **1c** (2- and 3-hydroxypropanal, respectively). They are both characterized by a moderately large dipole moment, and they have been experimentally characterized recently. However, the former is not confirmed in the ISM and the latter has been only tentatively detected.^21,26^

The six isomers lying above 3-hydroxypropanal (going to energies higher than 100 kJ/mol with respect to **2a**) are all diol species: **18** (1-propene-1,1-diol), **14** ((1*Z*)-propene-1,2-diol), **16** (2-propene-1,1-diol), **12** (2-propene-1,2-diol), **15** ((1*E*)-propene-1,2-diol), and **11** ((1*Z*)-propene-1,3-diol). Of these, **11**, **12**, **14,** and **15** have also been shown to form in interstellar ice analogues made of methanol and acetaldehyde by Wang et al.^35^ It is worth pointing out that other enol species, such as vinyl alcohol and (*Z*)-1,2-ethenediol, whose formation has been predicted by similar laboratory experiments,^72–76^ have been found in the ISM.^71,77–79^ This makes these diol species promising candidates for future radioastronomical searches, also considering that high energy isomers are present in the ISM when resulting from gas-phase mechanisms.^8,80^ However, suitable precursors are needed to produce such unstable/reactive species in experimental set-ups.^81^ According to previous studies, methylmalonic acid might be used to obtain 1-propene-1,1-diol.^82^

As mentioned above, Step 4 of our computational protocol provides all the parameters required for the rotational spectroscopic characterization of the 15 most stable C_3_H_6_O_2_ species. Therefore, our computed rotational parameters can be compared to those experimentally available. In particular, as already mentioned, the rotational spectrum has been recorded and analyzed for cis-propanoic acid (**2a**), methyl acetate (**3a**), *trans*- and *gauche*-ethyl formate (**4a** and **4b**), 1-hydroxyacetone (**5a**), 2-hydroxypropanal (**6d**), and 3-hydroxypropanal (**1c**). Three of these molecules are characterized by an internal methyl rotation, thus the comparison between the experimental and computed values is not straightforward and, indeed, it has been restricted only to rotational constants. For cis-propanoic acid, the parameters derived from the fit based on unresolved transitions recorded by Jaman et al.^24^ were considered. For **3a**, the parameters obtained in the principal-axis-method (PAM) system by Tudorie et al.^83^ have been used, while for 1-hydroxyacetone, the computed values were compared to the spectroscopic parameters derived by Apponi et al.^84^ after the diagonalization of the rotational tensor. This set of rotational constants is extended by consideration of the species without internal rotation: (i) both conformers of ethyl formate (**4a** and **4b**), (ii) 2- and 3-hydroxypropanal (**6d**, **1c**), and (iii) glycidol (**30**). Altogether, the molecules mentioned above provide a solid set of reference for benchmarking the outcomes of our protocol for the C_3_H_6_O_2_ family. For the last set of species, the comparison has also been extended to the centrifugal distortion constants and, whenever experimental data are available, to the molecular dipole moment.

Table 2 reports the comparison between computed and experimental ground-state rotational constants for the molecules detailed above. Inspection of this table points out that, on average, the absolute error on the rotational constants is of about 0.098%, in line with what expected from the literature.^86^ The maximum absolute deviation (∼0.2%) is noted—as expected—for the *A* rotational constant. If molecules without internal rotational are not considered in the analysis, the absolute error lowers to about 0.07%.

**Table 2:** Comparison between Computed and Experimental Ground-State Rotational Constants (in MHz) for *cis*-Propanoic Acid (**2a**), Methyl Acetate (**3a**), *trans*- and *gauche*-Ethyl Formate (**4a** and **4b**), 1-Hydroxyacetone (**5a**), 2-Hydroxypropanal (**6d**), 3-Hydroxypropanal (**1c**), and Glycidol (**30**)

	*B*_0_ ^a^	Exp.^b^	Δ%^c^	Δ*B*_vib_ ^a^
2a	10157.788	10155.358(2)^24^	+0.02	–89.241
	3820.219	3817.887(1)^24^	+0.06	–36.269
	2877.169	2875.174(1)^24^	+0.07	–23.796
				
3a	10256.079	10227.36(40)^83^	+0.28	–66.205
	4170.997	4164.544(55)^83^	+0.15	–46.824
	3078.173	3073.112(46)^83^	+0.16	–31.051
				
4a	17779.725	17746.68029(297)^85^	+0.19	–135.424
	2906.405	2904.733015(169)^85^	+0.06	–30.274
	2580.788	2579.148290(182)^85^	+0.06	–23.836
				
4b	9996.573	9985.58184(298)^85^	+0.11	+8.201
	3841.493	3839.60988(99)^85^	+0.05	–70.647
	3214.899	3212.86762(87)^85^	+0.06	–46.042
				
5a	10099.772	10074.875(51)^84^	+0.25	–63.407
	3811.927	3817.2550(90)^84^	–0.14	–43.641
	2865.063	2866.6157(100)^84^	–0.05	–30.755
				
6d	8443.812	8433.07428(18)^26^	+0.13	–80.858
	4238.844	4237.233774(64)^26^	+0.04	–42.898
	3146.43	3145.213978(77)^26^	+0.04	–35.734
				
1c	8659.629	8657.60135(34)^21^	+0.02	–29.067
	4117.977	4116.27295(17)^21^	+0.04	–81.911
	3128.173	3125.63135(11)^21^	+0.08	–41.775
				
30	10365.331	10347.86551(10)^d^	+0.17	–70.09
inner	4099.42	4102.38046(3)^d^	–0.07	–66.457
	3779.743	3781.93269(3)^d^	–0.06	–55.251
				
30	13888.334	13857.0864(2)^d^	+0.23	–55.576
outer	3418.266	3420.49834(5)^d^	–0.07	–49.351
	3065.88	3065.88426(5)^d^	–0.01	–39.876

a From CCSD­(T)/CBS+CV equilibrium rotational constants corrected for revDSD/junTZ vibrational contributions.

b Uncertainties are reported in parentheses in units of the last digit.

c Values obtained in this work.

d Δ % is the signed relative deviation.

Moving to the quartic centrifugal distortion terms, the comparison between theory and experiment has been performed for 6 species out of 9 (for the reasons explained above) and it is reported in Table S5 of the SI. For quartics, the mean absolute error considering 30 different values is 5.2%. For glycidol, the absolute error is on average 4.1% and 1.6% for inner and outer, respectively. For 2- and 3-hydroxypropanal, the errors are slightly larger, these being 5.1% and 7.2%. Instead, deviations around 8–9% are observed for the conformers of ethyl formate. These slightly larger uncertainties can be explained by invoking perturbations due to accidental degeneracies between rotational levels of the ground and low-lying vibrational states.^85^

As far as the sextic terms are concerned, the set of available data consists of 38 constants and the averaged deviation is about 20%. The computed values always agree in sign with the experimental ones, but they appear over- or underestimated without a specific trend. Such discrepancies are slightly larger than those affecting the quartic centrifugal distortion constants, but it has to be noted that sextic terms are small parameters and their experimental determination is very sensitive to the set of constants actually used in the fit.

The last parameter addressed is the dipole moment. In this regard, experimental measurements are available only for glycidol, ethyl formate, and propanoic acid. For glycidol and ethyl formate, the comparison is reported in the SI and shows that the experimental and computed values agree in the order of 0.08 debye, thus well within the uncertainty of the level of theory. For these species, the largest deviation is always for the μ_*c*_ component. Interestingly, for propanoic acid, the computed data are 0.187 D and 1.535 D for μ_*a*_ and μ_*b*_, respectively. These perfectly match the experimental derived data of 0.19 D and 1.54 D.^68^ This points out the reliability of the dipole moments computed in the present study for the isomers lying within the 150 kJ/mol range from the most stable species (Step 3).

The uncertainties derived above for the computed rotational parameters can be assumed valid for all the members of the C_3_H_6_O_2_ family and, as such, applied to the prediction for those species for which no experimental measurements have been reported. The spectroscopic parameters of potential candidates for future works are reported in Table S6 of the SI. For them, conservative errors of 0.1% for the rotational constants, 6% for the quartic centrifugal distortion constants, and 25% for the sextic terms can be considered.

Taken together, our findings demonstrate that our computational protocol is effective in providing accurate and predictive rotational spectroscopic data. Its dual nature is able not only to accurately exploit the MEP and indicate which isomers and/or conformers should be prioritized in laboratory studies, but also provide accurate spectral simulations to guide experiments. Overall, the proposed computational protocol: (i) provides the first exhaustive characterization of the isomers and conformers of the C_3_H_6_O_2_ family, thus pointing out that previous studies only showed the tip of the iceberg; (ii) extends the spectroscopic characterization of new C_3_H_6_O_2_ species by providing accurate computational data with well-defined uncertainties; (iii) establishes a solid basis for guiding future laboratory experiments, which in turn are a key step toward radioastronomical searches.

## Supplementary Material









## Notes

Approximate elapsed time for each step of the protocol for 2-hydroxypropanal (2a) and inner glycidol; label, structure, and relative energy (with and without hZPE correction) for the C_3_H_6_O_2_ species considered in the preliminary investigations (Step 1 and Step 2 of the protocol); figure showing the energy ranking of the 97 conformers at the B3/junDZ level as obtained from Step 2; relative energies (with and without hZPE correction) and total electric dipole moments (debye) for the C_3_H_6_O_2_ conformers considered in Step 3 of the protocol; experimental spectroscopic parameters (A-reduction) of inner and outer glycidol from this work and from Marstokk et al.;^34^ comparison between computed and experimental quartic and sextic centrifugal distortion constants for six species of the C_3_H_6_O_2_ isomer family (PDF)
All the CCSD­(T)/CBS+CV geometries in internal coordinates (ZIP)
SPFIT/SPCAT files of inner glycidol (ZIP)
SPFIT/SPCAT files of outer glycidol (ZIP)
