# Supplementary material for: Energetic and Spectroscopic Insights into the C3H6O2 Isomer Family for Astrochemical Purposes
Source: ACS Earth Space Chem. 2025 Dec 16;10(1):198–209. doi: 10.1021/acsearthspacechem.5c00291 (PMC12814775; doi:10.1021/acsearthspacechem.5c00291)
Supplement: Supplementary file 1 [file sp5c00291_si_001.pdf]

Supporting information for: "Energetic and Spectroscopic Insights into the $C_3H_6O_2$ Isomer Family for Astrochemical Purposes"

Alessandra Savarese,† Silvia Alessandrini,*,† Mattia Melosso,† Gabriele Panizzi,† Michela Nonne,‡ Luca Bizzocchi,† and Cristina Puzzarini*,†

†*Dipartimento di Chimica "Giacomo Ciamician", Università di Bologna, Via P. Gobetti 85, 40129 Bologna, Italy*

‡*Scuola Superiore Meridionale, Largo San Marcellino 10, 80138 Naples, Italy*

E-mail: silvia.alessandrini7@unibo.it; cristina.puzzarini@unibo.it

Table S1: Elapsed time [a] for each step of the protocol for 2-hydroxypropanal (**2a**) and `inner` glycidol.

**Elapsed time**		**Unit**	**Step**
2-hydroxypropanal (**2a**)	`inner` glycidol		
8	8	seconds	1-2
5	1	hours	3
27	91	days	4

[a] Calculations performed on an AMD EPYC 7282 16-Core Processor CPU, on nodes with 256 GB of RAM.

Table S2: Label, structure, and relative energy (with and without hZPE correction) for the $C_3H_6O_2$ species considered in the preliminary investigations (Steps 1 and 2 of the protocol).

LABEL	STRUCTURE	NAME	E_{el} [a]	E_{el}+hZPE [a]
**2a**	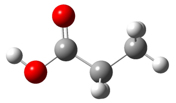	propanoic acid	0	0
**2c**	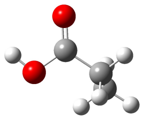		4.91	5.50
**2b**	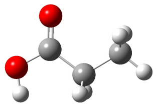		22.49	21.79
**2d**	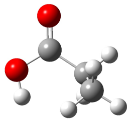		28.46	28.14
**3a**	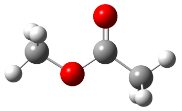	methyl acetate	39.40	36.65
**4b**	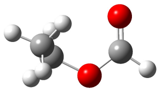	ethyl formate	55.08	55.32
**4a**	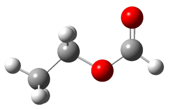		56.04	55.45

continues on the next page

LABEL	STRUCTURE	NAME	E_{el} [a]	E_{el}+hZPE [a]
**3b**	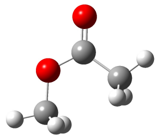		69.23	66.52
**4c**	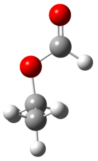		73.69	72.07
**5a**	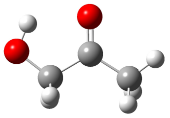	1-hydroxyacetone	87.43	83.38
**5c**	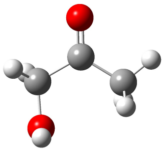		103.96	99.60
**5d**	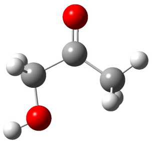		105.09	99.98
**6d**	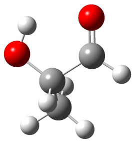	2-hydroxypropanal	106.49	103.15
**1c**	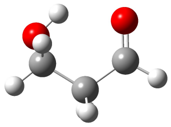	3-hydroxypropanal	112.92	112.12

continues on the next page

LABEL	STRUCTURE	NAME	E_{el} [a]	E_{el}+hZPE [a]
**5b**	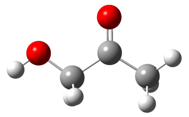		117.70	112.28
**6b**	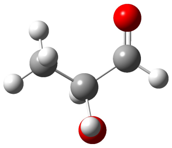		119.18	114.92
**18b**	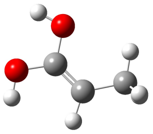	1-propene-1,1-diol	119.95	115.89
**6a**	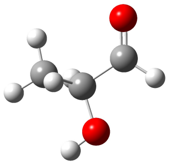		121.50	116.48
**18c**	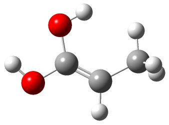		122.00	117.36
**6c**	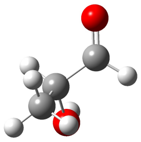		122.52	118.01
**18d**	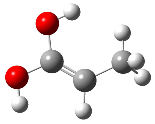		125.62	122.17

continues on the next page

LABEL	STRUCTURE	NAME	E_{el} [a]	E_{el}+hZPE [a]
**1a**	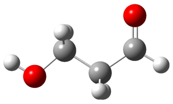		127.33	124.39
**1b**	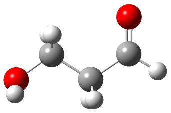		128.21	125.41
**18a**	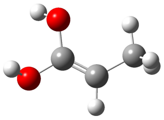		129.68	126.41
**1h**	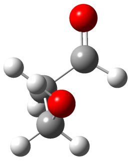		129.69	127.07
**6e**	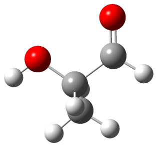		132.83	128.07
**1i**	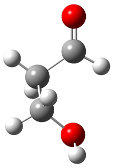		132.02	129.00
**1l**	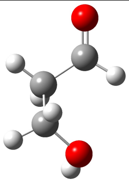		131.76	129.22

continues on the next page

LABEL	STRUCTURE	NAME	E_{el} [a]	E_{el}+hZPE [a]
**1g**	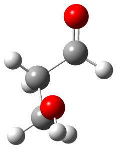		132.51	129.28
**1d**	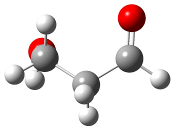		133.08	130.08
**1f**	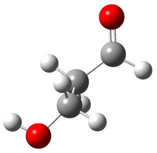		133.71	131.33
**1m**	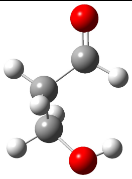		134.85	131.75
**1e**	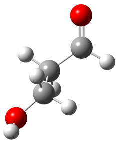		136.57	133.35
**14a**	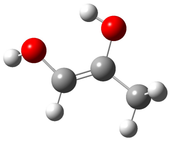	(1Z)-propene-1,2-diol	136.41	133.98
**14b**	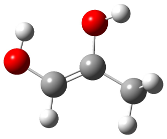		138.88	136.49

continues on the next page

LABEL	STRUCTURE	NAME	E_{el} [a]	E_{el}+hZPE [a]
**11b**	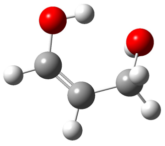	(1Z)-propene-1,3-diol	145.28	147.90
**16d**	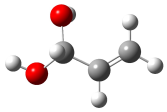	2-propene-1,1-diol	149.16	148.27
**16e**	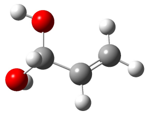		149.09	148.28
**12c**	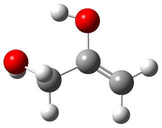	2-propene-1,2-diol	149.01	149.48
**15b**	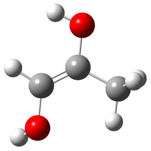	(1E)-propene-1,2-diol	153.68	149.62
**15d**	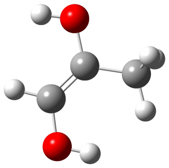		154.01	149.83
**13a**	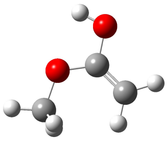	1-methoxy eth-1-enol	154.52	152.04

continues on the next page

LABEL	STRUCTURE	NAME	E_{el} [a]	E_{el}+hZPE [a]
**11e**	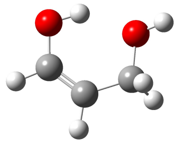		151.25	152.32
**12f**	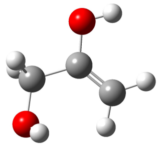		154.31	153.14
**15a**	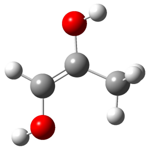		158.24	153.27
**16a**	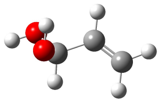		155.89	153.85
**16f**	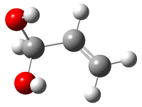		155.92	154.71
**15c**	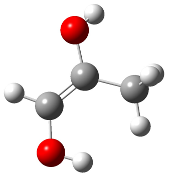		158.87	154.75
**12d**	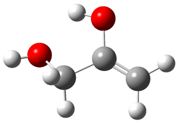		155.85	155.15

continues on the next page

LABEL	STRUCTURE	NAME	E_{el} [a]	E_{el}+hZPE [a]
**12h**	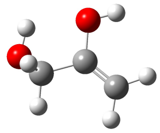		156.80	155.56
**13d**	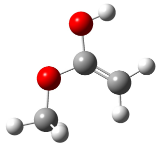		159.88	157.89
**14c**	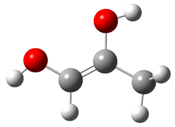		163.47	157.93
**16h**	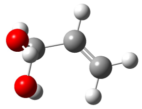		161.33	159.61
**12e**	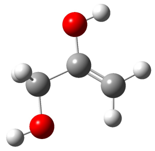		162.51	159.77
**16b**	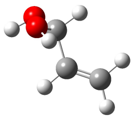		163.28	160.33
**25a**	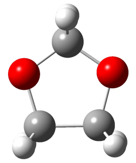	1,3-dioxolane	155.27	161.09

continues on the next page

LABEL	STRUCTURE	NAME	E_{el} [a]	E_{el}+hZPE [a]
**12g**	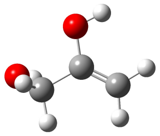		162.58	161.27
**7b**	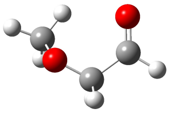	methoxyacetaldehyde	165.63	161.39
**16c**	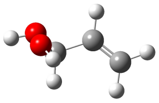		164.71	161.47
**7c**	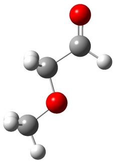		167.39	161.54
**12b**	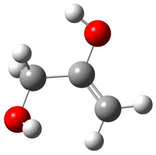		163.61	161.58
**7d**	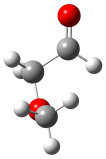		167.25	162.66
**13b**	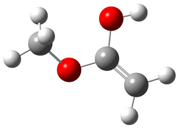		167.06	163.61

continues on the next page

LABEL	STRUCTURE	NAME	E_{el} [a]	E_{el}+hZPE [a]
**16l**	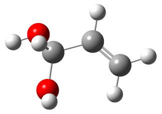		167.02	164.31
**10h**	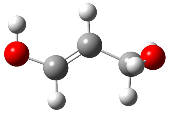	(1E)-propene-1,3-diol	164.86	164.74
**16g**	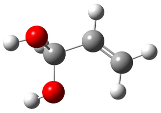		167.88	164.81
**16i**	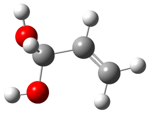		169.94	166.24
**10f**	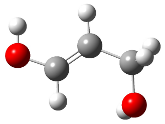		167.55	167.59
**11g**	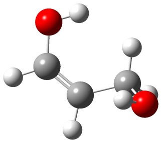		167.91	168.02
**7a**	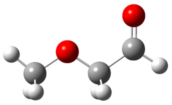		173.53	168.45

continues on the next page

LABEL	STRUCTURE	NAME	E_{el} [a]	E_{el}+hZPE [a]
**12i**	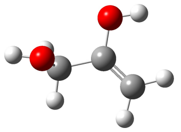		171.14	168.45
**13c**	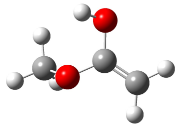		172.66	169.16
**12a**	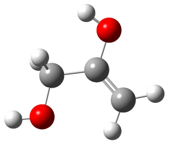		174.77	170.58
**10c**	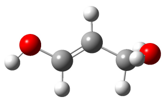		173.82	172.56
**11c**	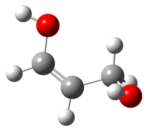		173.14	172.57
**10e**	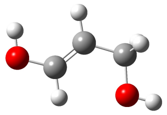		174.20	172.71
**11a**	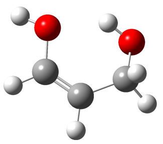		173.68	173.01

continues on the next page

LABEL	STRUCTURE	NAME	E_{el} [a]	E_{el}+hZPE [a]
**19f**	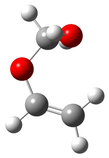	ethenyloxy methanol	172.91	173.03
**10g**	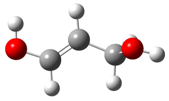		174.42	173.13
**10b**	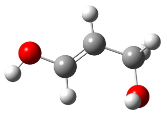		174.63	173.68
**19b**	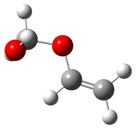		176.14	174.80
**11f**	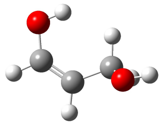		176.89	176.00
**19e**	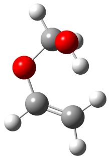		176.32	176.65
**10a**	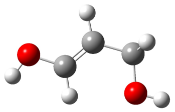		180.32	177.91

continues on the next page

LABEL	STRUCTURE	NAME	E_{el} [a]	E_{el}+hZPE [a]
**19d**	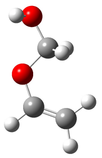		179.37	178.85
**36b**	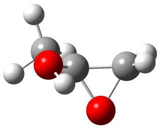	glycidol	182.41	179.29
**32a**	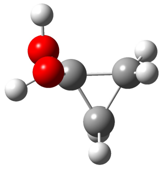	cyclopropane-1,1-diol	180.27	179.31
**11d**	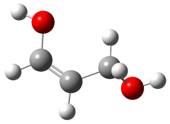		181.87	179.95
**29a**	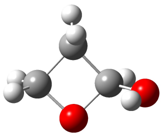	oxetan-2-ol	177.86	180.85
**10d**	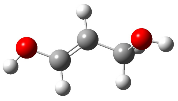		183.70	181.24
**19c**	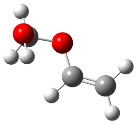		183.87	182.11

continues on the next page

LABEL	STRUCTURE	NAME	$\mathbf{E_{el}}$ [a]	$\mathbf{E_{el}}$+hZPE [a]
**34b**	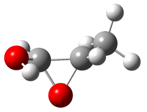	3-methyl-oxiran-2-ol	185.83	183.68
**29b**	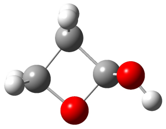		181.66	184.27
**19a**	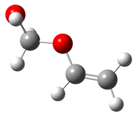		187.66	185.15
**32b**	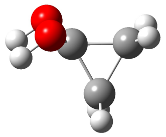		191.82	189.78
**36a**	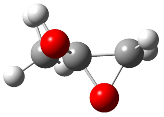		194.09	190.39
**34a**	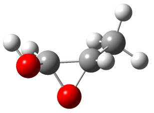		198.20	194.53
**22**	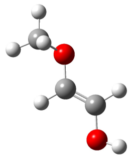	(E)-2-methoxy ethenol	212.69	208.38

continues on the next page

LABEL	STRUCTURE	NAME	E_{el} [a]	E_{el}+hZPE [a]
**23**	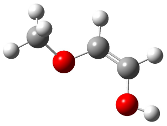	(Z)-2-methoxy ethenol	213.55	209.92
**35**	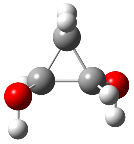	cyclopropane-1,2-diol	221.65	220.18
**27**	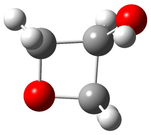	oxetan-3-ol	219.00	220.80
**30**	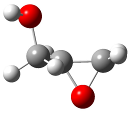	2-oxiranylmethanol	237.52	236.82
**33**	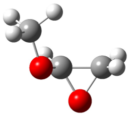	2-methoxyoxirane	249.04	247.06
**26**	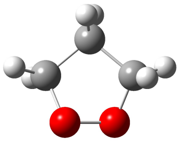	1,2-dioxacyclopentane	343.74	346.75
**31**	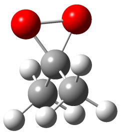	3,3-dimethyldioxirane	354.31	347.63

continues on the next page

LABEL	STRUCTURE	NAME	E_{el} [a]	E_{el}+hZPE [a]
**17**	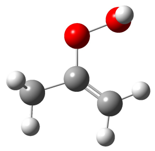	2-hydroperoxyprop-1-ene	370.49	362.69
**38**	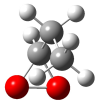	ethyl dioxirane	380.59	377.19
**21**	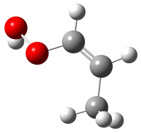	1-hydroperoxy-(1Z)-propene	388.08	380.60
**20**	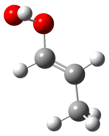	1-hydroperoxy-(1E)-propene	389.79	381.57
**9**	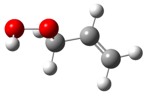	3-hydroperoxyprop-1-ene	410.33	403.36
**24**	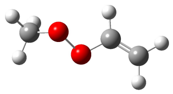	methylperoxy ethene	412.64	404.25
**28**	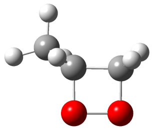	3-methyl-1,2-dioxetane	416.71	414.63

continues on the next page

LABEL	STRUCTURE	NAME	E_{el} [a]	E_{el}+hZPE [a]
**37**	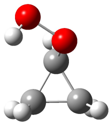	1-hydroperoxy cyclopropane	431.87	426.62

[a] The electronic energy E_{el} and the hZPE correction are computed at the B3LYP/cc-pVDZ level of theory. The energy of **2a** is taken as reference (equilibrium energy = -268.4277894 E_h and hZPE correction = 0.0900259 E_h). The values reported are in kJ/mol.

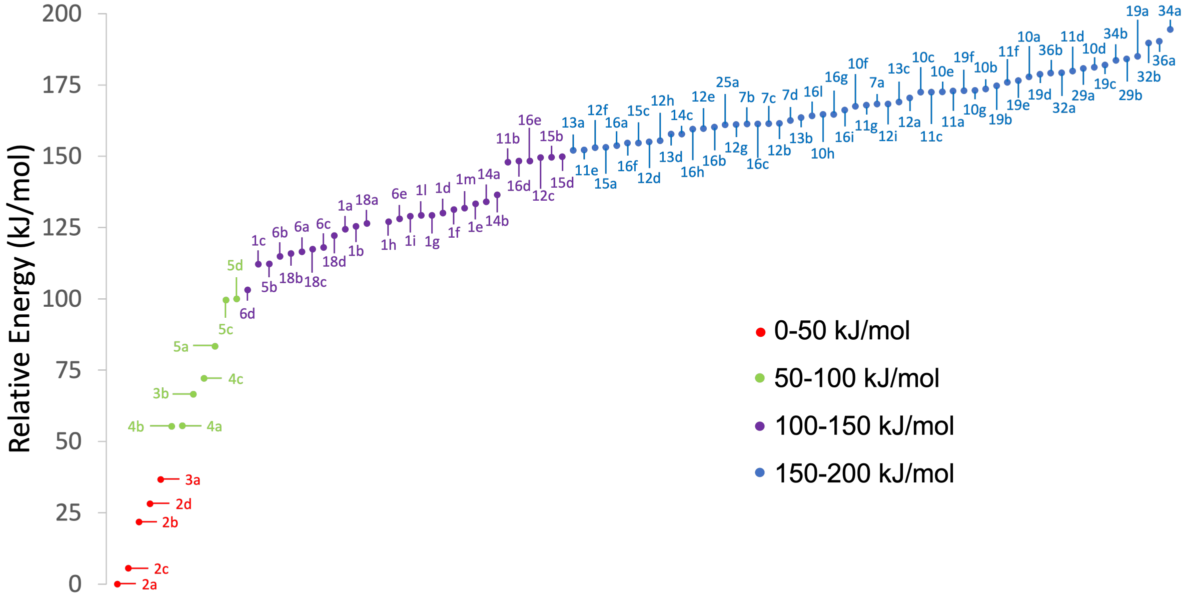

Figure S1: B3LYP/cc-pVDZ hZPE-corrected relative energies of the 97 $C_3H_6O_2$ conformers considered in Step 2. The energy of **2a** is taken as reference (equilibrium energy = -268.427789 E_h and hZPE correction = 0.090026 E_h).

Table S3: Relative energy (with and without hZPE correction) and total electric dipole moment (debye) for the $C_3H_6O_2$ conformers considered in Step 3 of the protocol.

ISOMER	E_{el} [a]	E_{el}+hZPE [a]	Total Dipole Moment [b]
**2a**	0	0	1.57
**2c**	3.59	4.32	1.81
**2b**	20.25	19.77	4.22
**2d**	25.50	25.42	4.44
**3a**	41.53	39.71	1.83
**4a**	57.91	58.01	2.13
**4b**	58.60	59.26	1.92
**3b**	72.00	70.01	4.55
**4c**	77.21	76.03	4.42
**5a**	83.02	79.59	3.22
**5d**	97.80	93.49	2.81
**5c**	99.54	95.54	2.12
**6d**	101.51	98.47	2.60
**5b**	108.39	103.56	4.36
**1c**	109.71	108.88	3.02
**6a**	114.00	109.19	2.89
**6c**	113.51	109.47	1.49
**6b**	114.78	110.42	1.56
**18b**	114.82	111.19	1.31
**18c**	117.34	113.10	1.66
**1a**	118.45	115.92	1.25
**1g**	121.25	118.51	4.36
**1h**	121.04	118.61	3.13

continues on the next page

ISOMER	E_{el} [a]	E_{el}+hZPE [a]	Total Dipole Moment [b]
**18d**	121.65	118.61	2.22
**1b**	121.71	118.99	2.83
**6e**	124.20	119.70	4.32
**1i**	122.05	119.71	4.44
**18a**	124.46	121.23	1.31
**1l**	124.52	121.99	3.35
**1e**	124.64	122.10	3.02
**1f**	124.67	122.46	1.33
**1d**	125.70	123.00	4.11
**14a**	114.82	125.32	2.06
**14b**	130.89	128.18	2.75
**16e**	136.07	135.06	0.20
**16d**	136.28	135.21	0.15
**12c**	141.26	141.02	1.65
**15b**	147.34	143.27	1.96
**15d**	147.34	144.75	0.51
**11b**	144.54	146.70	2.05

[a] The electronic energy E_{el} and the hZPE correction are computed at the revDSDPBEP86/jun-cc-pVTZ level of theory. The energy of **2a** is taken as reference (electronic energy = -268.0460105 E_h and hZPE correction = 0.0908268 E_h). The values reported are in kJ/mol.

[b] The total dipole moment is computed at the revDSDPBEP86/jun-cc-pVTZ level of theory. The values reported are in debye.

Table S4: Experimental spectroscopic parameters (A-reduction) of the `inner` and `outer` conformers of glycidol.

		`inner`		`outer`	
Constant	Unit	This work[a]	Marstokk et al.[1]	This work[a]	Marstokk et al.[1]
A_0	MHz	10347.8659(1)	10347.8575(1)	13857.0862(2)	13857.0779(4)
B_0	MHz	4102.36084(4)	4102.35728(4)	3420.50500(5)	3420.50399(9)
C_0	MHz	3781.95191(4)	3781.94826(4)	3065.87772(5)	3065.8781(1)
Δ_J	kHz	2.38385(2)	2.38257(5)	2.33346(3)	2.3412(3)
Δ_{JK}	kHz	-1.43220(8)	-1.4269(3)	-15.2339(2)	-15.156(3)
Δ_K	kHz	5.1842(3)	5.1812(1)	54.8963(8)	54.852(2)
δ_J	kHz	0.314837(7)	0.315582(9)	0.40322(1)	0.40240(9)
δ_K	kHz	-9.7037(2)	-9.782(1)	3.3009(6)	3.184(8)
Φ_J	mHz	-2.175(5)	-4.03(2)	5.467(8)	10.532(3)
Φ_{JK}	mHz	11.26(6)	16.60(2)	-62.0(1)	-
Φ_{KJ}	mHz	-30.6(2)	-104.4(4)	118.5(5)	237.4(2)
Φ_K	mHz	39.2(4)	77.2(2)	122.7(8)	-
ϕ_J	mHz	-0.300(2)	-	1.407(3)	-
ϕ_{JK}	mHz	69.16(4)	-	-23.9(3)	-
ϕ_K	mHz	-438.(2)	-	438.(3)	-
Λ_J	µHz	-0.0044(4)	-	0.0232(7)	-
Λ_{JJK}	µHz	0.376(2)	-	-0.386(7)	-
Λ_{JK}	µHz	-4.72(1)	-	-1.81(7)	-
Λ_{KKJ}	µHz	10.93(5)	-	-2.5(3)	-
Λ_K	µHz	-6.5(1)	-	-	-
λ_J	µHz	0.0017(1)	-	0.0078(3)	-
λ_{JK}	µHz	-	-	0.36(3)	-
Lines[b]		3748/5932		2795/4853	
Max. J, K_a		99, 46		89, 39	
rms	kHz	39.5		39.9	
St. Dev.		0.98		1.04	

[a] The standard errors as provided by `PIFORM` (Z. Kisiel, PROSPE - Programs for ROtational SPEctroscopy, `http://info.ifpan.edu.pl/~kisiel/prospe.htm`) are indicated in parentheses. Experimental dipole moment values are from Marstokk et al.[1].
[b] Distinct frequencies included in the fit / Total number of transitions. Our dataset includes 73 transitions for `inner` and 66 for `outer` from Marstokk et al.[1].

Table S5: Comparison between computed [a] and experimental [b] quartic and sextic centrifugal distortion constants for `inner` and `outer` glycidol, *trans* and *gauche* ethyl formate, and 2- and 3-hydroxypropanal.

**Constant**	**Unit**	**`inner` glycidol**		**`outer` glycidol**		**2-hydroxypropanal**	
		Exp.[c]	Theo.	Exp.[c]	Theo	Exp.[d]	Theo
D_J / Δ_J	kHz	2.50514(1)	2.54	2.30591(3)	2.30	1.784714(65)	1.79
D_K / Δ_K	kHz	5.7911(2)	5.79	54.7590(8)	53.83	17.82873(73)	18.54
D_{JK} / Δ_{JK}	kHz	-2.16004(7)	-2.35	-15.0687(2)	-15.05	-2.09014(21)	-2.41
d_1 / δ_J	kHz	-0.314786(3)	-0.325	-0.403240(10)	-0.40	0.304926(10)	0.31
d_2 / δ_K	kHz	0.060684(1)	0.056	-0.013772(3)	-0.01	2.57516(14)	2.47
H_J / Φ_J	Hz	-2.537(1)$\times10^{-3}$	-2.8$\times10^{-3}$	5.498(8)$\times10^{-3}$	6.9$\times10^{-3}$	0.004466(23)	0.0047
H_K / Φ_K	Hz	24.0(1)$\times10^{-3}$	26.6$\times10^{-3}$	0.0964(8)	0.065	0.1122(10)	0.087
H_{JK} / Φ_{JK}	Hz	7.11(2)$\times10^{-3}$	8.7$\times10^{-3}$	-0.07388(7)	-0.091	-0.00694(10)	-0.0096
H_{KJ} / Φ_{KJ}	Hz	-11.02(8)$\times10^{-3}$	-14.8$\times10^{-3}$	0.1576(4)	0.2368	-0.9838(52)	-0.087
h_1 / ϕ_J	mHz	-0.2984(3)	-0.383	1.395(3)	1.85	0.8170(20)	0.93
h_2 / ϕ_{JK}	mHz	0.1607	0.139	-0.017(1)	-0.013	8.712(46)	8.90
h_3 / ϕ_K	mHz	0.01296(7)	0.009	0.01155(7)	0.012	5.61(23)	3.97
μ_a	D	0.61(2)	0.63	1.25(6)	1.31		
μ_b	D	1.20(9)	1.23	1.650(1)	1.51		
μ_c	D	0.51(12)	0.78	0.154(2)	0.19		

**Constant**	**Unit**	***trans* ethyl formate**		***gauche* ethyl formate**		**3-hydroxypropanal**	
		Exp.[d]	Theo	Exp.[d]	Theo	Exp.[d]	Theo
D_J / Δ_J	kHz	0.6243410(238)	0.622	5.93910(91)	5.61	4.010060(69)	3.84
D_K / Δ_K	kHz	51.3539(120)	48.7	78.7548(167)	-69.3	12.99819(71)	11.5
D_{JK} / Δ_{JK}	kHz	-3.48608(285)	-3.60	-32.3373(38)	-29.40	-9.53279(46)	-8.58
d_1 / δ_J	kHz	0.1017021(129)	0.104	2.00186(36)	1.87	1.274532(30)	1.21
d_2 / δ_K	kHz	-0.9852(33)	-0.634	7.1777(187)	6.62	5.30738(43)	5.04
H_J / Φ_J	Hz	-	5.37$\times10^{-5}$	-0.05934(40)	-0.052	-0.019624(13)	-0.016
H_K / Φ_K	Hz	-	1.19	7.650(43)	5.46	0.25706(76)	0.21
H_{JK} / Φ_{JK}	Hz	-	0.307$\times10^{-3}$	0.9168(39)	0.67	0.12908(25)	0.11
H_{KJ} / Φ_{KJ}	Hz	-0.8331(174)	-0.737	-4.1566(119)	-3.05	-0.28906(53)	-0.26
h_1 / ϕ_J	mHz	-	0.029	-25.608(175)	-22.5	-9.1793(59)	-7.55
h_2 / ϕ_{JK}	mHz	-24.11(103)	-4.71	-73.4(112)	-127.7	-61.90(11)	-43.7
h_3 / ϕ_K	mHz	-209.(43)	-210.6	1785.(42)	548.7	-81.47(63)	-50.1
μ_a	D	1.85(2)	1.94	1.44(6)	1.55		
μ_b	D	0.69(8)	0.74	1.05(3)	1.05		
μ_c	D	0[e]	0.0	0.25	0.25		

[a] Computed at the revDSD/junTZ level of theory. [b] The experimental references are taken from this work for glycidol, from ref. 2 for 2-hydroxypropanal, ref. 3 for both isomers of ethyl formate and ref. 4 for 3-hydroxypropanal. [c] Watson's S reduction. [d] Watson's A reduction. [e] Assumed in ref. 5.

Table S6: Computed vibrational ground state rotational constants, [a] quartic and sextic centrifugal distortion terms, [b] and electric dipole moment [b] of the most stable $C_3H_6O_2$ species without experimental spectroscopic characterization (**2c**, **2b**, **2d**, **3b**, **4c**, **5d**, **5c**, and **5b**).

**Constant**	**Unit**	**2c**	**2b**	**2d**	**3b**	**4c**	**5d**	**5c**	**5b**
A_0	MHz	8765.46	10001.51	8596.98	8998.87	17316.55	8618.53	8521.05	9948.93
B_0	MHz	3944.39	3830.59	3944.63	4391.62	2658.67	4087.15	4006.09	3728.44
C_0	MHz	3135.87	2869.75	3102.28	3065.45	2535.42	2878.21	2858.11	2808.87
D_J	kHz	2.15	0.727	1.938	0.625	2.95	0.694	0.774	0.652
D_K	kHz	−132.1	2.81	−116.2	−0.170	814.7	−0.171	−0.703	6.54
D_{JK}	kHz	164.1	3.45	145.1	5.25	−70.9	5.20	7.39	3.84
d_1	kHz	0.202	−0.193	−0.0175	−0.208	−0.830	−0.233	−0.181	−0.173
d_2	kHz	−0.0375	−0.0192	−0.0164	−0.0485	−0.0407	−0.0643	−0.106	−0.0268
H_J	mHz	−86.5	0.335	−76.7	−0.0686	−33.5	0.541	0.902	−0.0407
H_K	Hz	12.4	0.0223	11.6	−0.00113	372.9	−0.0182	0.276	0.0381
H_{JK}	mHz	1373.0	−19.6	1316.0	−5.12	1517.7	−25.3	4.40	−5.14
H_{KJ}	Hz	−7.22	0.0125	−7.646	0.00983	−36.9	0.0506	−0.356	−0.0145
h_1	mHz	11.9	0.0607	11.4	0.0601	−12.3	−0.0444	−0.517	0.0673
h_2	mHz	25.8	−0.122	22.9	0.0211	0.314	−0.308	0.102	0.101
h_3	mHz	6.22	0.000747	5.11	−0.0540	0.101	0.0403	−0.121	−0.00450
$\|\mu_a\|$	D	1.26	1.68	3.78	3.22	3.97	2.80	1.57	0.08
$\|\mu_b\|$	D	1.26	3.87	1.90	3.22	0.23	0.14	1.11	4.36
$\|\mu_c\|$	D	0.33	0.00	1.36	0.00	1.91	0.00	0.89	0.14
μ_{tot}	D	1.81	4.22	4.44	4.55	4.42	2.81	2.12	4.36

[a] CCSD(T)/CBS+CV equilibrium rotational constants augmented by revDSD/junTZ vibrational contributions. [b] Computed at the revDSD/junTZ level of theory.

References

(1) Marstokk, K. M.; Møllendal, H.; Stenstrøm, Y. Microwave spectrum of oxirane methanol (glycidol), the assignment of a second hydrogen-bonded conformer and conformational composition in the gas phase and in solution. *Acta Chem. Scand.* **1992**, *46*, 432–441, DOI: 10.3891/acta.chem.scand.46-0432.

(2) Alonso, E. R.; McGuire, B. A.; Kolesniková, L.; Carroll, P. B.; León, I.; Brogan, C. L.; Hunter, T. R.; Guillemin, J.; Alonso, J. L. The laboratory millimeter and submillimeter rotational spectrum of lactaldehyde and an astronomical search in Sgr B2(N), Orion-KL, and NGC 6334I. *Astrophys. J.* **2019**, *883*, 18, DOI: 10.3847/1538-4357/ab3463.

(3) Medvedev, I. R.; De Lucia, F. C.; Herbst, E. The Millimeter-and Submillimeter-Wave Spectrum of the Trans and Gauche Conformers of Ethyl Formate. *Astrophys. J. Suppl. Ser.* **2009**, *181*, 433.

(4) Fried, Z. T. P.; Motiyenko, R. A.; Sanz-Novo, M.; Kolesniková, L.; Guillemin, J.-C.; Margulès, L.; Uhlíková, T.; Belloche, A.; Jørgensen, J. K.; Holdren, M. S.; Xue, C.; Štěpán Urban; Jiménez-Serra, I.; Rivilla, V. M.; McGuire, B. A. Rotational spectroscopy and tentative interstellar detection of 3-hydroxypropanal ($HOCH_2CH_2CHO$) in the G+0.693-0.027 molecular cloud. 2025; https://arxiv.org/abs/2508.15911.

(5) Riveros, J. M.; Wilson Jr, E. B. Microwave spectrum and rotational isomerism of ethyl formate. *J. Chem. Phys.* **1967**, *46*, 4605–4612.
